# Photodegradation and photocatalysis of per- and polyfluoroalkyl substances (PFAS): A review of recent progress

**DOI:** 10.1016/j.nxmate.2023.100077

**Published:** 2024-01

**Authors:** Sanny Verma, Bineyam Mezgebe, Charifa A. Hejase, Endalkachew Sahle-Demessie, Mallikarjuna N. Nadagouda

**Affiliations:** a Pegasus Technical Services INC., Cincinnati, OH 45219, USA; b Groundwater Characterization and Remediation Division, Center for Environmental Solutions and Emergency Response, US EPA, Ada, OK 74820, USA; c Department of Chemical and Environmental Engineering, University of Cincinnati, Cincinnati, OH 45221, USA; d Land Remediation and Technology Division, Center for Environmental Solutions and Emergency Response, US EPA, Cincinnati, OH 45268, USA; e Water Infrastructure Division, Center for Environmental Solutions and Emergency Response, US EPA, Cincinnati, OH 45268, USA

**Keywords:** PFAS, Advanced oxidation technologies, Photodegradation, Photocatalysis

## Abstract

Per- and polyfluoroalkyl substances (PFAS) are oxidatively recalcitrant organic synthetic compounds. PFAS are an exceptional group of chemicals that have significant physical characteristics due to the presence of the most electronegative element (i.e., fluorine). PFAS persist in the environment, bioaccumulate, and have been linked to toxicological impacts. Epidemiological and toxicity studies have shown that PFAS pose environmental and health risks, requiring their complete elimination from the environment. Various separation technologies, including adsorption with activated carbon or ion exchange resin; nanofiltration; reverse osmosis; and destruction methods (e.g., sonolysis, thermally induced reduction, and photocatalytic dissociation) have been evaluated to remove PFAS from drinking water supplies. In this review, we will comprehensively summarize previous reports on the photodegradation of PFAS with a special focus on photocatalysis. Additionally, challenges associated with these approaches along with perspectives on the state-of-the-art approaches will be discussed. Finally, the photocatalytic defluorination mechanism of perfluorooctanoic acid (PFOA) and perfluorooctanesulfonic acid (PFOS) following complete mineralization will also be examined in detail.

## 1. Introduction

The extensive use of per- and polyfluoroalkyl substances (PFAS) in aqueous film-forming foams (AFFFs), non-stick technologies, and a variety of other coatings and industrial products has led to their widespread contamination in aqueous environments [1–9]. These organic compounds are universally found and dispersed in the aquatic environment across their life cycle through manufacturing, across the supply chain, product use, end-use, and manufacturing materials [10–15]. Consequently, PFAS have been introduced into aquatic environments from various non-point sources. The most significant volume of emitted PFAS (~95%) is directly released into the marine environment or ends in aquatic media as part of their fate and transport [16]. Thus, PFAS pose a major threat to drinking water supplies due to their associated ecological and human health risks.

PFAS, especially perfluorooctanesulfonic acid or perfluorooctane sulfonate (PFOS) and perfluorooctanoic acid (PFOA), have been widely detected in the blood of humans and wild animals as well as the environment [17–20]. These compounds have been extensively used in commercial and industrial applications as coatings for textiles, paper products, and cookware surface treatment and to formulate some firefighting foams for almost 60 years. After careful evaluation of the toxicological effects of these compounds, the application and manufacture of longer-chain PFAS have been limited [16,21].

In 2016, the U.S. Environmental Protection Agency (US EPA) issued a lifetime health advisory for PFOA and PFOS, which was further revised to a near-zero standard in 2023 [22–26]. However, several countries have continued manufacturing and using PFAS, contributing to additional environmental pollution with high concentrations of these compounds [27,28]. At environmentally relevant pH values, PFAS are organic anions and tend to be mobile in groundwater [29]. An extensive database of toxicity information is available for some other PFAS, but the toxicity of PFOA and PFOS remains the most studied [30–32].

**Fig. 1.**
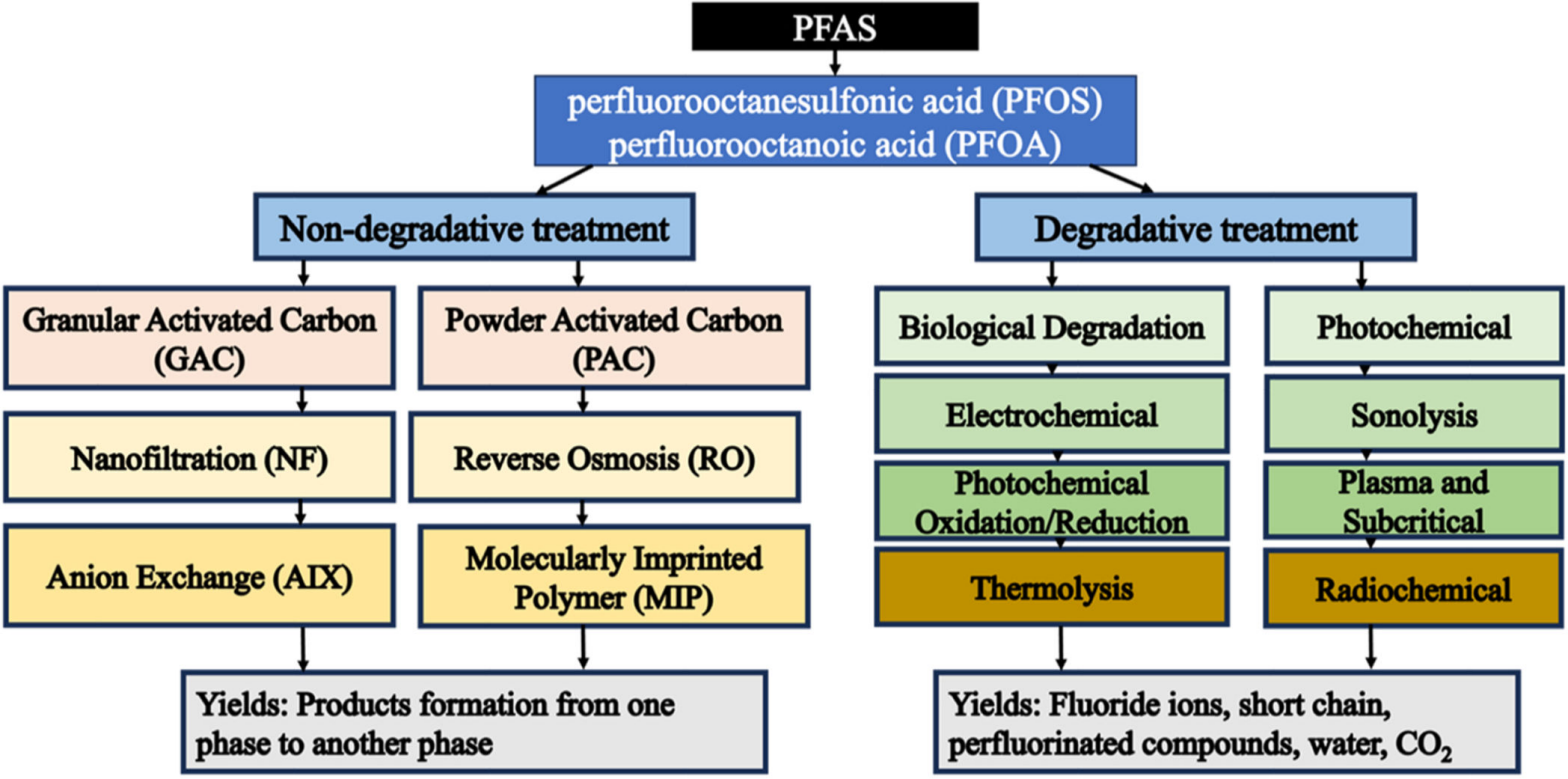
Overview of the state-of-the-art technologies employed for the degradation of PFOA and PFOS.

Various methods have been used to separate PFAS from contaminated water and degrade these substances into harmless and environmentally friendly products. Treatment technologies span a wide range of in-situ processes, non-destructive methods, ex-situ separation, or destructive methods [33]. Physical adsorption processes, including granular activated carbon (GAC), powdered activated carbon (PAC), anion exchange (AIX), molecularly imprinted polymers (MIP), and biomaterials, have been evaluated for the removal of PFAS from various complex water matrices. High-pressure-driven membrane processes (e.g., nanofiltration (NF) and reverse osmosis (RO)) have also been investigated (Fig. 1) [34]. NF and RO processes have shown to be effective in removing a broad range of PFAS, but their wide implementation has been hampered by cost and membrane fouling. AIX has shown higher adsorption capacity than GAC and PAC [35,36]. However, AIX is costly, making it economically infeasible for a large-scale application. Additionally, due to the slow dispersal of PFOA and PFOS in the pores of porous materials, AIX has shown a slow adsorption rate [37]. Moreover, the presence of other constituents (e.g., natural organic matter (NOM)) in surface waters can significantly lower the adsorption capacity [38].

More importantly, activated carbon, ion-exchange resins, and reverse osmosis are separation processes that generate secondary waste (i.e., concentrated waste that requires secondary treatment). Adsorption processes require regenerating the spent adsorbents after the adsorption bed breakthrough using thermal desorption, or require fresh adsorbent [39–42]. Recently, several promising PFAS degradation technologies, such as biological degradation [43] as well as advanced oxidation technologies (AOPs), including photochemical, sonolysis, electrochemical, thermolysis, chemical oxidation and reduction, plasma, subcritical and radiochemical treatment have been explored for the complete mineralization of PFAS [44]. Among these degradative technologies, the photochemical process showed more PFAS dissociation and defluorination at ambient reaction conditions. Previous studies have suggested that photocatalysis is a promising technology for the degradation of PFAS [45].

This paper provides a critical review of recent investigations on various aspects of PFAS photodegradation with a particular emphasis on photocatalysis. Additionally, challenges with these approaches along with the mechanisms associated with the photocatalytic dissociation of PFAS will be critically evaluated.

## 2. Catalyst-free photodegradation of PFAS using ultraviolet (UV) light

Continued exposure of organic compounds such as PFAS to radiation with wavelengths less than 320 nm, known as actinic wavelengths, results in photodissociation via photolysis. In a challenge to eliminate PFAS accumulation in the environment, various studies have examined their possible UV degradation into harmless species under mild reaction conditions. In photolysis, the dissociation of organic pollutants is motivated by the adsorption of photons, thus providing a new reaction pathway via the formation of electronically excited reactive species [46, 47]. Two forms of photolysis are capable of degrading PFAS: (1) Direct photolysis and (2) Indirect photolysis [48,49]. In direct photolysis of PFAS, photons are directly absorbed by the PFAS, causing it to undergo photodegradation. In fact, without using oxidants or photocatalysts, water is cleaved into hydrogen and hydroxyl radicals under UV radiation. For indirect photolysis (e.g., UV/H_2_O_2_, UV/Ozone, and photo-Fenton), separate compounds absorb photons (light energy/radiation) and then work as an intermediate to react with the contaminant (i.e., PFAS) [50,51].

**Scheme 1.**
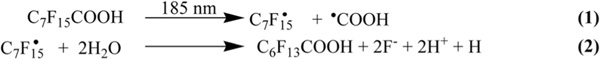
A plausible PFOA photodegradation mechanism at 185 nm UV irradiation [55].

In this regard, for a practicable photolysis process, the strong C-F bond in PFAS should be cleaved by UV irradiation into fluoride (F^−^) ions. In water, F^−^ ions can easily react with Ca^2+^ ions, forming an environmentally friendly compound such as inorganic calcium fluoride (CaF_2_) [52,53]. Jing *et al.* [54] reported an efficient degradation of PFOA (initial concentration: 25 parts per million (ppm)) in water (61.7%) into fluoride ions, perfluoroheptanoic acid, perfluorohexanoic acid, perfluoropentanoic acid, and perfluorobutanoic acid within two hours of 185 nm vacuum ultraviolet (VUV) light irradiation. This study proposed a mechanism for the degradation of PFOA through the successive loss of CF_2_ moieties at 185 nm VUV-irradiation. Initially, photodegradation of PFOA produced heptyl (${\text{C}}_{7}{\text{H}}_{15}^{•}$) and carboxyl (^•^COOH) free radicals (Scheme 1; Eq. 1). Next, in-situ generated heptyl radical reacts with H_2_O, resulting in the formation of perfluoroheptanoic acid (PFHpA) and F^−^ ions.

**Scheme 2.**
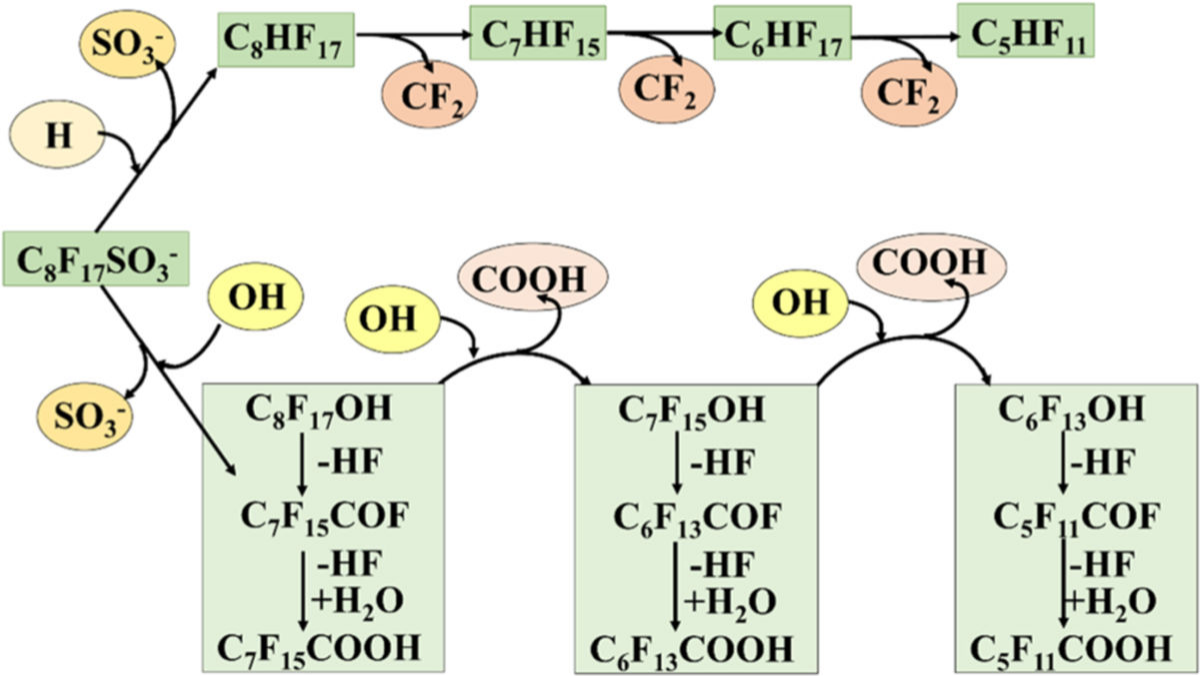
The plausible reaction mechanism for the photooxidation of PFOA [55].

Recently, Xin *et al.* [56] investigated the direct photolysis of 19 representative PFAS, including perfluorocarboxylic acids (PFCAs), fluorotelomer unsaturated carboxylic acids (FTUCAs), and hexafluoropropylene oxide dimer acid (GenX) under far-UVC 22 nm. PFCAs, FTUCAs, and GenX showed enhanced photolysis compared to the conventional 254 nm irradiation, where up to 81% of the parent decayed, and a defluorination of 31% was achieved within 4 h. In another study, Yamamoto *et al.* [55] studied the photodegradation of PFOS into fluoride, sulfate ions, and other short-chain fluorinated by-products in water and alkaline two-propanol solution. In the alkaline two-propanol solution, 76% and 92% PFOS (initial concentration: 40 μM) dissociation were achieved after one and ten days, respectively, when irradiated with 254 nm UV light using low-pressure 32 W mercury lamp. However, PFOS degradation efficiency in water was reduced to 8% and 68%, respectively, after one day and ten days of treatment. The first-order photodegradation rate constant of PFOS in alkaline two-propanol and water was reported as 0.93 days^−1^ and 0.13 days^−1^, respectively, confirming the solvent effect on the dissociation of PFOS. Based on these experimental studies, two plausible reaction pathways were proposed for the photodegradation of PFOS (Scheme 2). In one reaction pathway, PFOS (C_8_HF_17_SO_3_) was dissociated into C_8_HF_17_ via the cleavage of C-S bonds between C_7_F_15_ and ${\text{SO}}_{3}^{-}$ and the addition of proton. On the other hand, C_8_F_17_OH was proposed by the decomposition of the C-S bond and the addition of hydroxide. Next, C_8_HF_17_ and C_8_F_17_OH intermediates, which further dissociated into C_7_HF_15_ and C_7_F_15_OH, respectively, by stepwise elimination of CF_2_ unit (Scheme 2) [55].

Higher temperatures boost PFOS photodegradation via the reductive process due to creating a chemically favored local environment and the enhanced interfacial mass transfer at the gas-liquid interface [57]. The rate of PFOS dissociation increases with the increasing heating intensity under UV irradiation in the absence of photocatalyst. However, slow dissociation was reported under oxygenation and higher hydronium levels [57].

The effects of higher pH via a catalyst-free reductive route of PFOS showed a pseudo-first-order decomposition rate constant of 0.91 h^−1^ in an aqueous solution [58], two orders of magnitude higher than earlier studies [59]. Photolysis has limited effects on degrading PFAS under environmental conditions, but under higher VUV and UV energy, PFAS can be dissociated. Competitive UV irradiation absorption by NOM limits the rate of photolysis and photochemical degradation [60,61]. NOM primarily hinders photodegradation by consuming UV photon energy, acting as a scavenger for reactive species, including hydroxyl and peroxyl radicals and generating NOM-derived oxidative intermediates [61]. Table 1 reports a few non-catalytic photodegradation approaches for PFAS mineralization.

### 2.1. Photochemical mediators for PFAS dissociation under UV light irradiation

Photochemical oxidants, including persulfate, hydrogen peroxide, and ozone have been mostly used for recalcitrant organic pollutants [63–67]. In combination with UV light, these photochemical oxidants generate active radicals and react with organic compounds to degrade into unharmful compounds in water treatment [68–72]. In this section, we describe the role of photochemical oxidants, mainly phosphotungstic acid, and persulfate, in the mineralization of PFAS under UV irradiation (Table 2).

#### 2.1.1. Phosphotungstic acid

**Scheme 3.**
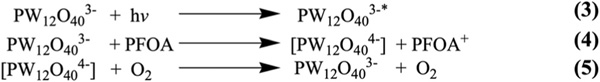
The reaction mechanism for the activation of phosphotungstic acid [53].

Phosphotungstic acid, H_3_PW_12_O_40_, is a polyoxometalate or heteropolyacid and has been studied as a mediator for the degradation of organic pollutants in water treatment [73–77]. At a pH= 2, H_3_PW_12_O_40_ produces ${\text{PW}}_{12}{\text{O}}_{40}^{3-}$, which absorbs UV light and is converted into a photoexcited state ${\left[{\text{PW}}_{12}{\text{O}}_{40}\right]}^{3-*}$. ${\left[{\text{PW}}_{12}{\text{O}}_{40}\right]}^{3-*}$ is considered the initiation step of photocatalysis (Scheme 3; Eq. 3) [78]. Next, an electron transfer from PFOA to ${\left[{\text{PW}}_{12}{\text{O}}_{40}\right]}^{3-*}$ leads to the formation of [PW_12_O_40_]^4−^, which is re-oxidized to [PW_12_O_40_]^3−^ in the presence of O_2_ (Eq. 5).

Eq. 4 expresses one-electron-oxidized PFOA undergoing C-C bond cleavage between C_7_F_15_ and COOH to yield C_7_F_15_ free radical and CO_2_. When PFOA (C_0_=1.35 mmol/L) reacted with H_3_PW_12_O_40_ (C=6.7 mmol/L) at pH < 2 under UV (Xe-Hg lamp) irradiation at 4.8 oxygen atm, only 50% photodegradation of PFOA was observed after 24 h.

Similarly, Hori *et al.* [53] reported the decomposition of non-afluoropentanoic acid (PFPA) into F^−^ and CO_2_ using water-soluble H_3_PW_12_O_40_ in aqueous solution at room temperature under UV-Vis irradiation in the presence of O_2_. The negative aspect of this type of treatment is the detection of high CF_4_ levels during the dissociation of PFAS by high energy procedures.

#### 2.1.2. Persulfate (${\text{S}}_{2}{\text{O}}_{8}^{2-}$)-induced oxidation for PFAS photochemical decomposition

Although persulfate ions (${\text{S}}_{2}{\text{O}}_{8}^{2-}$) is not a photocatalyst, it is an appealing substance to photochemically decompose PFAS as persulfate photolysis gives two sulfate radicals, ${\text{SO}}_{4}^{.-}$[79,80]. Sulfate radicals work as strong oxidant in aqueous solution (Eq. 6).

(6) $${\text{S}}_{2}{\text{O}}_{8}^{2-}+\text{h}\nu \to 2{\text{SO}}_{4}^{•-}$$

**Scheme 4.**
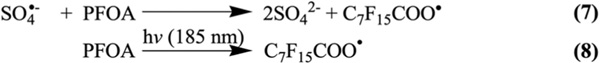
Activation of PFOA by $\text{SO}4^{.-}$ radical [82].

Hori *et al.* [81] showed that persulfate (${\text{S}}_{2}{\text{O}}_{8}^{2-}$) influenced the photochemical dissociation of PFOA and yielded 100% mineralization within 4 h of 254 nm UV light irradiation. In this process, PFOA reacted with ${\text{SO}}_{4}^{.-}$ (from Eq. 6) and was then converted into C_7_F_15_COO^•^ radicals (Eq. 7), which was further degraded into short-chain perfluorinated carboxylic acids. In another study, under 185 nm irradiation, PFOA was equally dissociated via direct photolysis and by ${\text{SO}}_{4}^{.-}$ (Scheme 4; Eq. 8) [82]. The dissociation rate constant was reported to be 1.8 times greater than that at irradiation under 254 nm. This investigation demonstrated that the dissociation efficiency of PFOA was enhanced under 185 nm compared to that of 254 nm irradiation technology.

It was also observed that the presence of Fe^2+^ accelerated the photodegradation of persulfate ions into sulfate radicals, ${\text{SO}}_{4}^{.-}$ (Eq. 9) [83]. A combination of Fe^2+^/UV with persulfate ions enhanced the mineralization of PFOA.

(9) $${\text{S}}_{2}{\text{O}}_{8}^{2-}+{\text{Fe}}^{2+}\to {\text{Fe}}^{3+}+{\text{SO}}_{4}^{•-}+{\text{SO}}_{4}^{2-}$$

In the presence of other organic compounds, the degradation of PFAS through persulfate/UV was inhibited [84]. Therefore, pretreatment by removing NOM from the water matrix is needed prior to degrading PFAS.

## 3. Photocatalytic degradation of PFAS

Photocatalysts present in water under UV light irradiation produce reactive species such as hydroxyl radicals (•OH) as well as other photochemically generated reactive intermediates [87]. These reactive species actively react with pollutants such as PFAS to degrade them into harmless intermediates. While a couple of review papers focused on the photocatalytic degradation of PFAS in solutions [88–90], this review covers the photodegradation pathways and catalytic performance as it relates to the photocatalyst (i.e., material).

Semiconductor photocatalysts absorb UV light to produce conduction band (CB) electrons (e^−^) and valence band (VB) holes (h^+^) to degrade PFOS through redox reaction. The reaction under UV light irradiation and at higher pH (pH=11.8) can also occur via charge-transfer-to-solvent to produce strongly oxidizing OH^•^. Then, ${\text{e}}_{\text{aq}}^{-}$ acts as the main reductive species and can break C-C and C-F bonds in PFOS, leading to the complete mineralization of PFAS. These results support Qu *et al.* findings that the concentration of hydrated electron increased with an increase in the initial pH and enhanced the defluorination of PFOA in the UV-KI system [91]. The reaction rate constant for PFOA degradation was reported as 0.0295 min^−1^ at pH=10, about 49 times higher than that at pH-5. Thus, initial pH conditions increased the concentration of hydrated electrons and supported the reductive mineralization of PFAS in a homogeneous catalytic system [92]. This section discusses the role of nanocomposites and microparticle-based photocatalysis.

### 3.1. Nanocomposite-based photocatalysis for PFAS mineralization

The C-F bond in PFAS compound is very strong and requires an innovative method to facilitate C-F cleavage by directing energy transfer or contact with reactive species to the C-F locus [87]. Semiconductor nano-photocatalysts, including titanium dioxide and nanostructured indium oxide, have been employed in water treatment applications [93]. The bandgap between the CB and VB of the material electrons plays a major role in the activation of a photocatalyst. The bandgap represents the minimum energy needed to stimulate an electron from the valance band to the conduction band and generates holes in the valance band. Further, these holes and electrons in combination generate reactive oxygen species such as superoxide (${\text{O}}_{2}^{-}$), and hydroxyl radical (OH^•^) [94]. The bandgap also depends on other factors, including size, structure, composition, and surface ligands of the nanocatalyst [95]. This section, provides literature survey of nano-enabled photodegradation technologies for treating PFAS is provided.

#### 3.1.1. Nano TiO_2_ and modified TiO_2_ photocatalysis

The photocatalytic degradation of PFOA using titanate nanotubes (TNTs) TiO_2_ P25 has been studied [96]. Direct photolysis (254-nm, 400 W UV lamp) of PFOA (C_0_=50 mg/L) gave the fastest 82% defluorination efficiency at pH=4, followed by 64% at pH=7 and then 56% at pH=10, resulting in fluorides and shorter chain perfluorocarboxylic acids (PFCA) after 48 h of UV irradiation. However, using TNTs as a photocatalyst showed 85%, 68%, and 55% defluorination of PFOA at pH=4, 7, and 10, respectively, after 24 h of irradiation. In this study, it was observed that TNTs were capable of eliminating PFOA and formed TNT-PFOA complexes. Furthermore, TNTs displayed better adsorption capacity compared to powder TiO_2_. This method demonstrated an adsorption capacity of 50 mg PFOA/g TNT at pH=4 for PFOA [96].

**Scheme 5.**
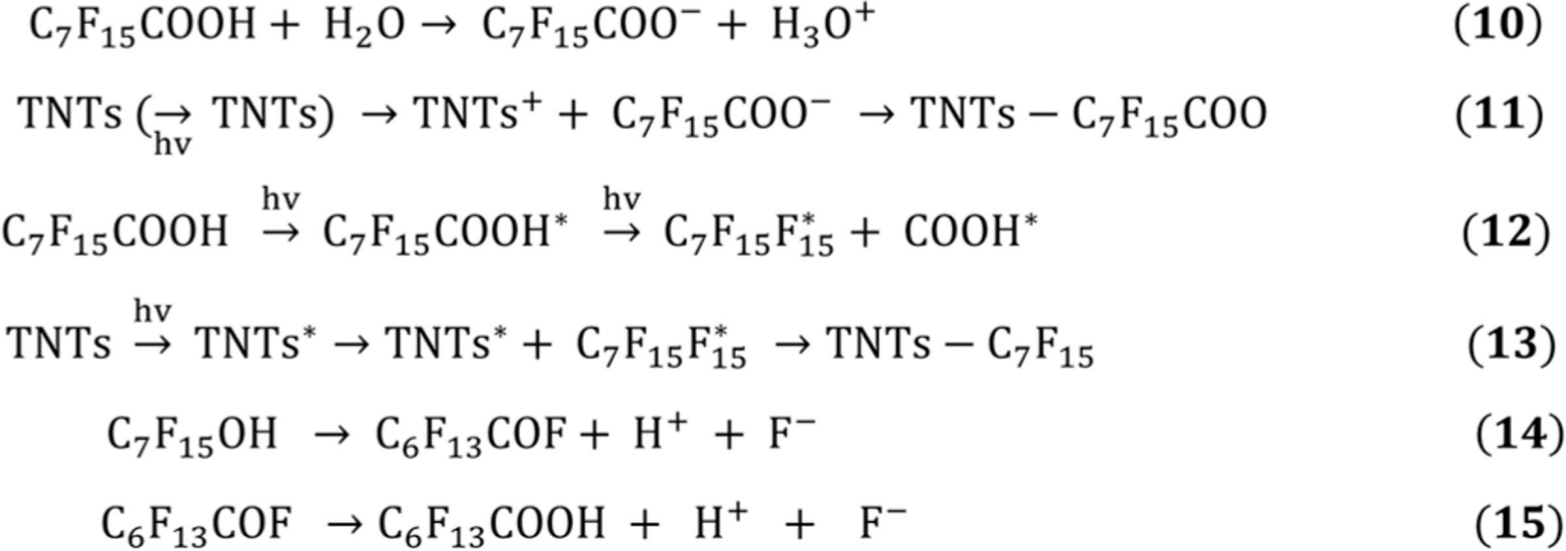
Proposed PFOA decomposition mechanism by TNTs [96].

A plausible reaction mechanism for the photodegradation of PFOA using TNTs under UV light is shown in Scheme 5. In aqueous media, PFOA exists in the anionic form, which can be adsorbed to the positively charged TNT to form TNTs-C_7_H_15_COO^−^ complex (Scheme 5; Eq. 10 and 11). As a result, unused PFOA is converted into an excited state of PFOA and then photolyzed into C_7_F_15_ and COOH^−^ radicals under UV irradiation (Scheme 5; Eq. 12). Further, ${\text{C}}_{7}{\text{F}}_{15}^{•}$ radical adsorbed to the surface of excited TNTs to form TNTs-C_7_F_15_ complex (Eq. 13). In addition, this intermediate radical (${\text{C}}_{7}{\text{F}}_{15}^{•}$) also reacted with water to form fluorinated alcohol (C_7_F_15_OH). In the next step, HF is eliminated from C_7_F_15_OH to form C_6_F_13_COF (Eq. 14), followed by the formation of C_6_F_13_COOH (PFHpA) with one less CF_2_ moiety (Eq. 15). Gatto *et al.* reported the complete mineralization of 4.0 mM PFOA when treated with TiO_2_ slurry (0.66 g/L) under UV irradiation of 95 W/m^2^ for 6 h [97]. The pseudo-first-order kinetic constant for PFOA photodegradation was reported as 0.1296 /h.

Adding a hole-scavenger (e.g., oxalic acid) also enhances PFOA photodegradation using TiO_2_ catalyst under a UV irradiation of 254 nm and nitrogen atmosphere. In contrast, adding an electron acceptor, such as potassium persulfate (K_2_S_2_O_8_) prevents the decomposition of PFOA [98]. In this case, oxalic acid, reactive carboxyl anion radical along with photogenerated electrons are formed, stimulating the photo-reductive decomposition of PFOA. Wang *et al.* [98] observed 10.5% and 12.4% of PFOA degradation in oxygen and nitrogen atmosphere, respectively, in the presence of TiO_2_ after 3 h of UV light irradiation. Following nitrogen purging of PFOA solution to remove dissolved oxygen, treatment with 3 mM oxalic acid (pH 2.47) resulted in 86.7% degradation. In contrast, the oxygenated solution of PFOA showed only 6.6% PFOA mineralization under the same reaction conditions.

Several TiO_2_ modifications have been established and explored to enhance the performance of PFAS dissociation. Tian *et al.* [99] used silver nanoparticles and molecularly imprinted polymers to modify TiO_2_ nanotubes (MIP-Ag/TiO_2_ NTs) for the photocatalytic degradation of PFOA into shorted chain fluorinated compounds. MIP-Ag/TiO_2_ NTs decomposed 91% of the PFOA within 8 h of irradiation and showed higher reactivity than other photocatalysts such as TiO_2_. The improved photocatalytic properties of MIP-Ag/TiO_2_ NTs can be attributed to footprint cavities created by molecularly imprinted polymer and electron traps of silver nanoparticles. Metal-doped titanium dioxide photocatalysts have also been investigated for adequate mineralization of PFAS.

Sansotera *et al*. [100] synthesized iron and niobium co-doped titanium dioxide (Fe:Nb-TiO_2_) via sol-gel method. Fe: Nb-TiO_2_ catalyst did not completely degrade PFOA, where only 14.8% degradation was achieved after 3 h at pH= 4.3. However, this catalyst demonstrated higher activity compared to commercially available TiO_2_ and un-doped TiO_2_. Later, Chen *et al.* reported the effective photocatalytic oxidative degradation of PFOA (initial concentration: 50 ppm) aqueous solution using transition metal (e.g., Cu and Fe) modified TiO_2_ (Cu-TiO_2_ and Fe-TiO_2_) catalyst. Among the different modified TiO_2_, Cu-TiO_2_ showed the highest reactivity. After 12 h of UV irradiation, Cu-TiO_2_ decomposed PFOA into fluoride ions (F-) and shorter perfluorinated carboxylic acids (e.g., C_6_F_13_COOH, C_5_F_11_COOH, C_4_F_9_COOH, C_3_F_7_COOH, C_2_F_5_COOH, and CF_3_COOH); yielding 91% decomposition and 19% defluorination. Other modified TiO_2_ photocatalysts such as noble metallic nanoparticles modified TiO_2_ (M-TiO_2_, M = Pt, Pd, Ag) [101], Pb-modified TiO_2_ (Pb-TiO_2_) [102] and composites TiO_2_ with multiple wall carbon nanotubes (MWCNTs) [103] have been investigated for the efficient and effective degradation of PFOA. These modified TiO_2_ heterogeneous photocatalysts yield traps to capture photo-induced electrons or holes, showing higher PFAS degradation compared to pure TiO_2_ or commercially available TiO_2_ P25 [102–104].

### 3.2. Alternative nano-enabled technologies for PFAS treatment

TiO_2_-based photocatalysts are effective in degrading most organic pollutants [104]. However, TiO_2_ has limited effectiveness in degrading PFOA [96–102,105,106]. Other nano-enabled AOPs have been explored to treat PFAS (Table 3). Electron-hole pair segregation and bandgap variation have effectively enhanced photocatalyst reactivity [107]. The introduction or immobilization of foreign metal on the surface of the photocatalyst/support can improve the electron-hole segregation. The usage of nanostructured metal oxides such as indium oxide (In_2_O_3_) with narrower bandgap, as well as nanoplates and porous microspheres with high surface area, can also improve the photocatalytic performance of photocatalysts [108].

**Fig. 2.**
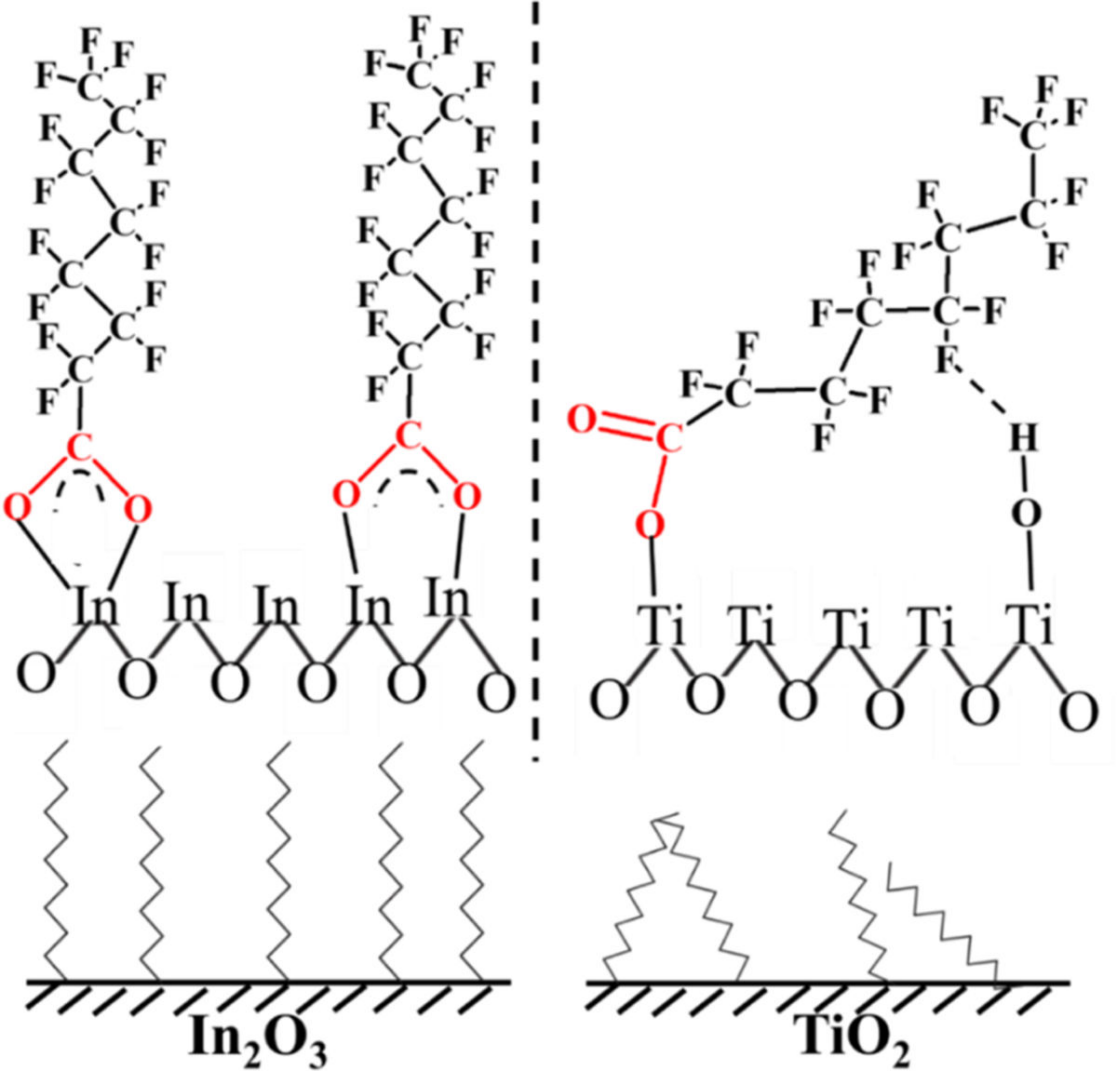
Schematic presentation of PFOA adsorbed onto In_2_O_3_ and TiO_2_ surfaces. Modified from [108].

**Fig. 3.**
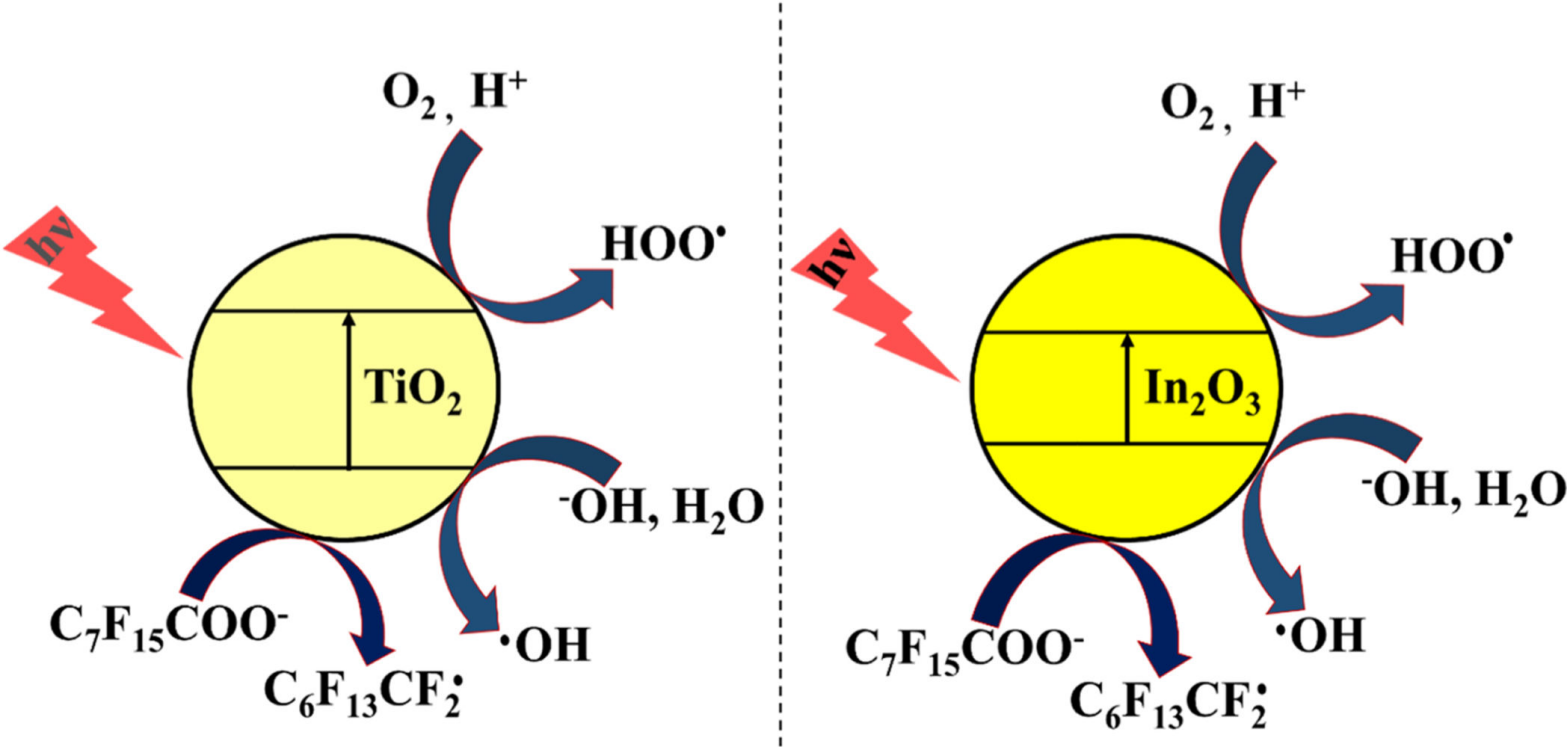
Plausible reaction mechanisms for the PFOA photodissociation on TiO_2_ and In_2_O_3_ surface. Modified from [108].

Li *et al.* [108] developed In_2_O_3_ as a photocatalyst for the efficient degradation of PFOA under UV irradiation with rate constant ~8.4 times higher than TiO_2_. Their findings suggest that the COO^−^ (carboxylate) functional group of PFOA strongly binds to the In_2_O_3_ surface via co-ordination bond in a bridging or bidentate arrangement, which is useful in the direct PFOA photodegradation under UV irradiation (Fig. 2). Solid-state fluorine-19 nuclear magnetic resonance (^19^ F Mass NMR) analysis of TiO_2_ confirmed the interaction of the inner CF_2_ group of PFOA with OH group of TiO_2_ surface via hydrogen bond (Fig. 2). PFOA molecules arrange onto the TiO_2_ surface in a monodentate manner, then photogenerated holes favorably convert to OH^•^. These hydroxyl radicals are less reactive toward PFOA in which it leads to minimized photodegradation of PFOA. Electron spin resonance (ESR) investigations also support the above statement regarding the coordination of PFOA to TiO_2_. Based on ESR studies, a plausible reaction mechanism of PFOA photodissociation on TiO_2_ and In_2_O_3_ surface has been proposed, as shown in Fig. 3. The proposed PFOA degradation mechanism consists of several stages. During the first step, perfluorinated alkyl radicals (C_7_F_15_COO^•^) are generated via electron transfer from COO^−^ to the photocatalyst (i.e., TiO_2_ and In_2_O_3_). In the next stage, perfluoroheptyl radical (${\text{C}}_{7}{\text{F}}_{15}^{•}$) forms via Kolbe decarboxylation reaction and is followed by ${\text{C}}_{7}{\text{F}}_{15}^{•}$ reacting with water to form an unstable alcohol (C_7_F_15_OH). C_7_F_15_OH is then converted to C_6_F_13_COF, which in turn reacts with water forming perfluoroheptanoic acid (C_6_F_13_COOH). In the following steps, short chain perfluorinated compounds (e.g., perfluoropentanoic acid (C_4_F_9_COOH)) are produced stepwise until complete mineralization is achieved. A detailed description of the proposed mechanism has been previously described by our group [109].

**Fig. 4.**
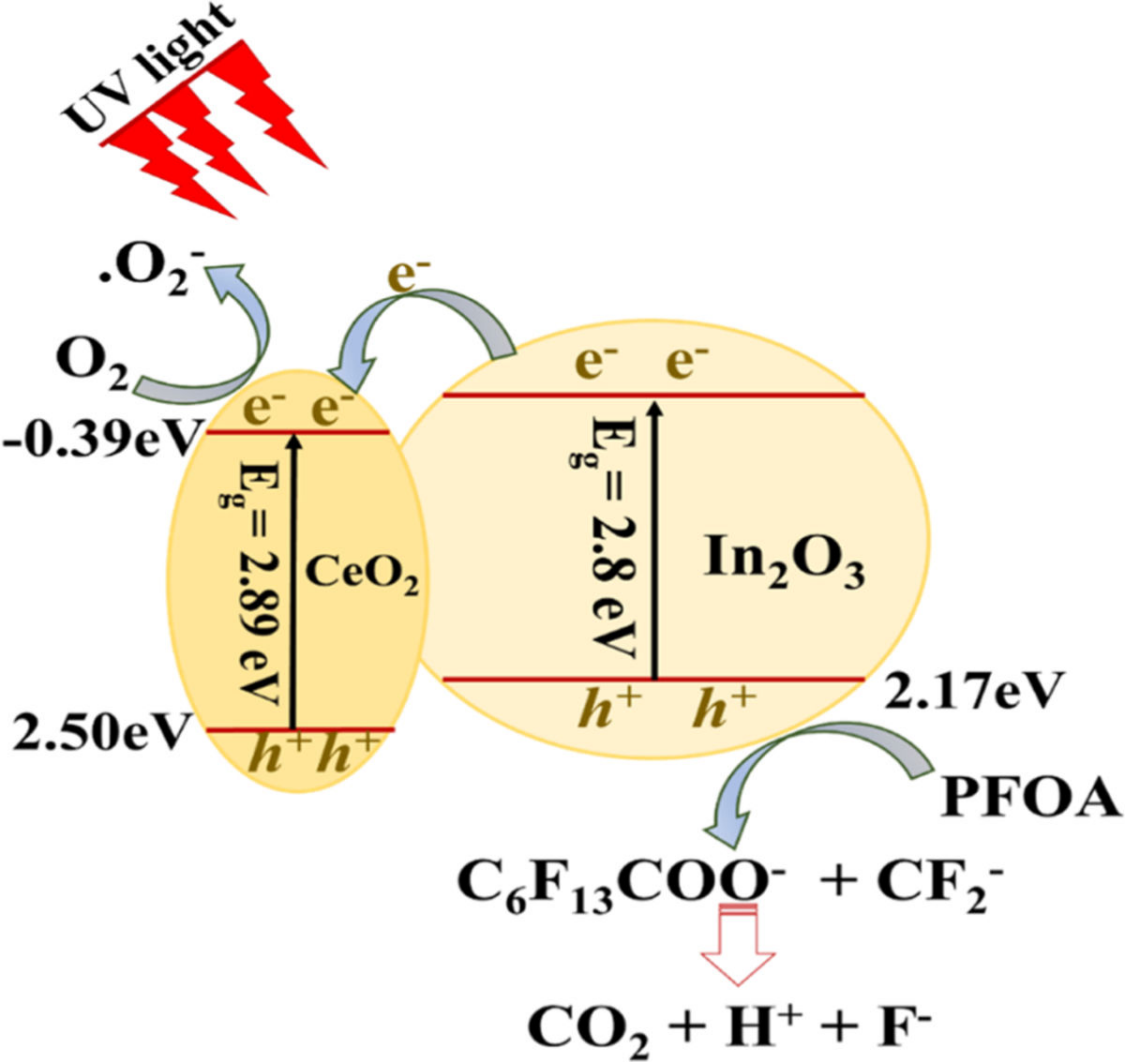
A proposed PFOA decomposition mechanism by CeO_2_/In_2_O_3_ under UV irradiation. Modified from [113].

Zhang’s research group further investigated other In_2_O_3_ based heterogeneous nano-photocatalysts such as In_2_O_3_ nanoporous, nanosphere (In_2_O_3_ NPNSs) [110], nanostructured In_2_O_3_ [111], In_2_O_3_-graphene nanocomposites [112] and CeO_2_-doped indium oxide (xCeO_2_/In_2_O_3_) with various CeO_2_ doping amounts [113] for the efficient photocatalytic mineralization of PFOA into non-toxic by-products (CO_2_ and fluoride ions) under UV light irradiation and mild reaction conditions (e.g., room temperature, weak acidic condition atmospheric pressure) (Fig. 4) (Table 4). Another needle-like nanostructured photocatalyst, gallium oxide (β-Ga_2_O_3_), has been reported to decompose PFOA via photo-reductive decomposition in surface or wastewater following first-order rate constants of 3.51 and 4.03 h^−1^, respectively [114]. In wastewater treatment and under UV irradiation of 185 nm, β-Ga_2_O_3_exhibited higher efficacy for the elimination of trace amounts of PFOA.

Other studies have reported photocatalysts that have been deactivated due to their interaction with natural organic compounds typically present in wastewater during PFOA treatment. In another study, Huang *et al.* [115] reported using SiC-graphene as a catalyst to degrade PFOA via hydrodefluorination process (HDF) under UV light (Scheme 5). Using SiC-graphene as a catalyst, PFOA photodegradation occurred by photo-stimulating electrons on SiC that rapidly transferred to the PFAS group through graphene surface resulting in a reduction in the electron cloud density of C-F bond [115].

**Scheme 6.**
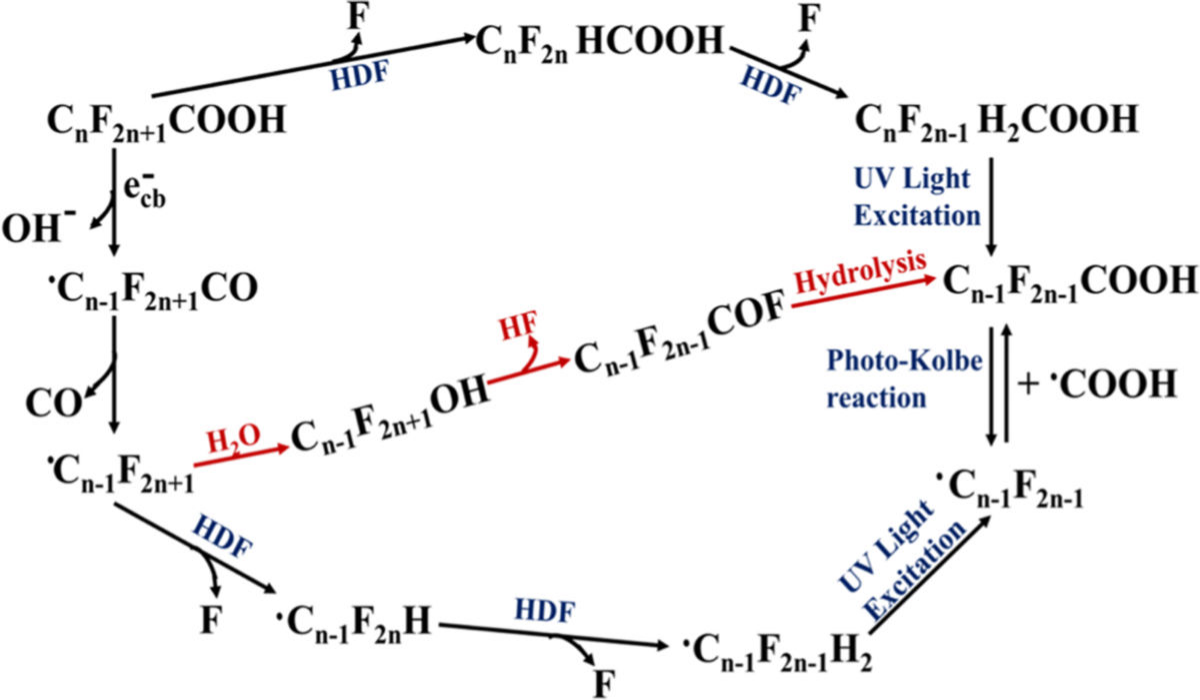
Possible hydrodefluorination mechanism for PFOA dissociation using SiC/Graphene as photocatalyst [115].

Studies have shown that the photodegradation of PFOA followed the hydrodefluorination mechanism through a two-step process that starts with reactive Si-H bonds forming on SiC-graphene surface under UV light irradiation. In the second step, F atoms at the α-position of the perfluoroalkyl group are substituted by hydrogen atoms (from Si-H) to form C_n_F_2n_HCOOH via the Si-H/C-F redistribution because of the nucleophilic substitution reactions (Scheme 5). C_n-1_F_2n-1_COOH is formed eliminating CH_2_ carbene from C_n_F_2n_HCOOH and the Photo-Kolbe decarboxylation reaction via carbon-carbon bond cleavage under UV light irradiation. C_7_F_15_ radical is formed by the reaction of PFOA with a photogenerated electron from the SiC-graphene catalyst, which can then be mineralized through the HDF step (Scheme 6) [115].

Huang *et al.* [116] also studied the effectiveness of photodecomposition of both branched and linear PFOS using superior photocatalyst SiC/graphene quantum dots (SiC-GQDs), where GQDs acted as an electron donor under 254 nm UV light irradiation [116]. It has been reported that PFOS was more challenging to degrade compared to PFOA.

GQDs were derived by oxidizing graphene using ultra-high frequency ultrasonication. This process is composed of SiC/GQDs nanocomposites, which involve the attachment of GQDs to SiC nanoparticles via a hydrothermal method. The synthesized material was tested for the photocatalytic degradation of PFOS. In this photoreaction, the photogenerated electrons (${\text{e}}_{\text{cb}}^{-}$) were generated from π−π * transition of C=C bond and the n−π * transition C=O bond. Next, because of the heterojunction structure of SiC/GQDs, these electrons were transported from the LUMO of GQDs to the CB of silicon carbide (SiC) nanoparticles. The ${\text{e}}_{\text{cb}}^{-}$ can be directionally transferred to the electron acceptor-PFOS, which is accumulated as a surfactant on the surface of the hydrophobic nanocomposite (SiC), resulting in the critical activation of -${\text{SO}}_{3}^{-}$ group. Next, PFOS was converted into ${\text{C}}_{\text{n}}{\text{F}}_{2\text{n}+1}^{•}$ free radicals and further dissociated to short-chain perfluorinated carboxylic acids via hydrolysis and hydrodefluorination [116].

Recently, Xu *et al.* [117] reported promising platinum-modified indium oxide nanorods (Pt/IONRs) photocatalysts for PFOA mineralization under UV light irradiation. Loading of Pt and the rod-like morphology of indium oxide promote light-harvesting, which further increases the charge carrier separation rate, enhancing of the photocatalytic ability of this catalyst. Along with the oxygen vacancies on the Pt/INORs surface, it also stimulated the photooxidation of PFOA [117].

In summary, heterogeneous nanocomposites photocatalysis is a good alternative for eliminating of PFAS under UV light irradiation (Table 4). However, further work is needed to establish the toxicity of the reaction by-products. The stability and leaching of the catalyst should also be determined before scaling-up the technology. Thus, the nanomaterials should be measured cautiously to ensure the safety of this technology. In natural water, to access the practicability of these technologies, the effect of dissolved organic matter such as humic acid, fulvic acid, and other coexisting ions (e.g., ${\text{CO}}_{3}^{2-}$, ${\text{SO}}_{4}^{2-}$, ${\text{NO}}_{3}^{-}$) should be evaluated.

### 3.3. Bismuth oxyhydroxphosphate (Bi_3_O(OH)(PO_4_)_2_) microparticle ultraviolet photocatalyst

Sahu *et al.* [118] recently studied the rapid degradation and mineralization of PFOA using bismuth oxyhydroxphosphate (Bi_3_O(OH) (PO_4_)_2_) microparticle as a photocatalyst under UV light irradiation. His research group also synthesized other reference catalysts such as sheaf-like β-Ga_2_O_3_ nanomaterial [119] and sub-micrometer particles BiPO_4_ photocatalysts [120] and tested for PFOA photodegradation. Compared to these reference catalysts, BOHP microparticles showed intensely quicker PFOA degradation and mineralization.

The rate constant for photocatalytic dissociation of PFOA by BOHP was approximately 15 times higher than both β-Ga_2_O_3_ and BiPO_4_ in the presence of NOM [121]. The BOHP photocatalyst was also examined at low PFOA concentrations and a rapid photocatalytic dissociation of PFOA was still achievable.

### 3.4. Photocatalytic degradation of PFAS via ozonation under UV light

**Scheme 7.**
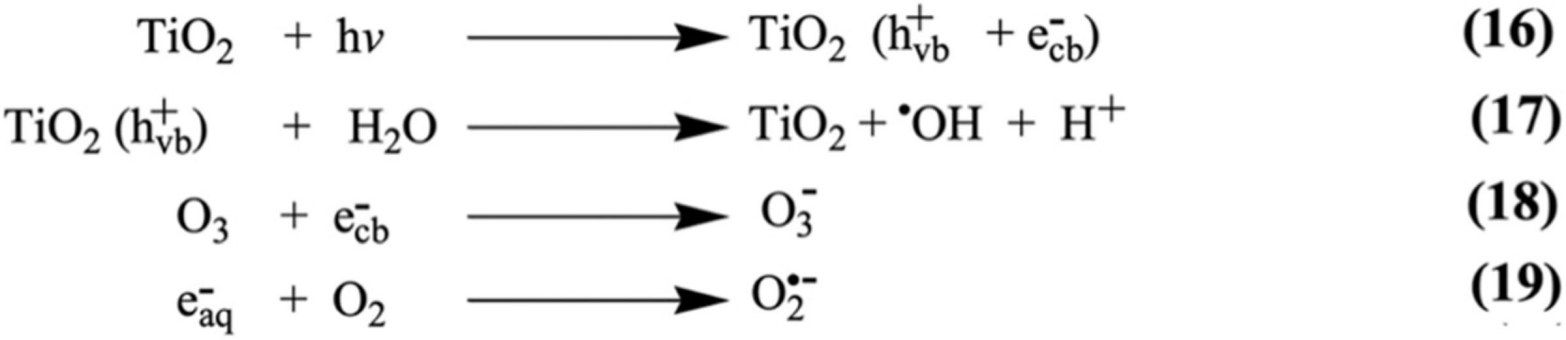
Formation of reactive species in the photocatalytic ozonation [127].

Ozone (O_3_) gas has been employed for the photodegradation of organic contaminants, PFOA, and PFOS [122–126]. Recently, Huang *et al.* [127] reported the efficient degradation of PFOA by photocatalytic ozonation using TiO_2_ and UV light irradiation. Li’s experiments showed that photocatalytic ozonation boosted the degradation efficiency of PFOA remarkably, which established the existence of the synergistic effect between ozonation and photocatalysis. The defluorination ratio of PFOA in UV/TiO_2_/O_3_ was found to be 4.18, which was 3.01 times higher than UV/TiO_2_/O_2_ and UV/O_3_ within 4 h of UV light irradiation. O_3_ is a stronger oxidizing agent than oxygen since it has high oxidizing ability (2.07 eV) and can easily react with a photogenerated electron to form reactive species ${\text{O}}_{3}^{-}$ (Scheme 7; Eq. 18).

Hence, the recombination of holes and photogenerated electrons decreased, leading to the enhanced PFOA photooxidative efficiency. In the presence of air, PFOA showed prolonged reductive degradation due to available reactive hydrated electrons (${\text{e}}_{\text{aq}}^{-}$) (generated from the photolysis of water) (Eq. 3) reacting with dissolved oxygen rapidly. Therefore, PFOA can be effectively photodegraded in the absence of O_2_ [128]. However, a recent study by Lashuk *et al.* [129] reported that photocatalytic ozonation inefficiently degraded PFAS with WO_3_/TiO_2_ under UVA-visible radiation, where the degradation efficiency was comparable to photocatalysis.

## 4. Summary

PFAS are concerning compounds due to their bioaccumulative and persistence abilities. PFOA and PFOS are the main types of PFAS, which have been broadly detected in wildlife, humans, and the environment. Due to the strong C-F bond, these highly persistent perfluorinated chemicals are not easily degraded into unharmful compounds via biodegradation and hydrolysis in complex environmental matrices. Various degradative technologies such as electrochemical degradation, sonolysis, chemical redox, thermal degradation, and photolysis have been evaluated for PFAS elimination. In this review, photocatalytic degradation has shown promise in the effective mineralization of PFAS in aqueous media under UV light irradiation. PFAS treated with UV/photocatalyst undergoes photo-reduction or photooxidation to yield short-chain organic compounds, F-, CO_2_, etc.

Most photodegradation studies have been performed using synthetic PFOA solutions. Additional studies using actual complex wastewater containing NOM/other dissolved organic species are needed to thoroughly evaluate the process at a larger scale. In this regard, the present review also provides detailed information on the plausible PFOA and PFOS photodegradation mechanisms, which can assist researchers in establishing and designing the process scale-up.

State-of-the-art approaches have primarily employed heavy metals to develop photocatalysts with high electron conductivity, resistivity, and large surface area. The toxicity and scarceness of these metals call for environmentally friendly materials that can exhibit promising photocatalytic performance. To enhance the photoactivity of photocatalysts, future research should focus on the synthesis of diverse photocatalyst, which can also harvest energy under a broader visible light spectrum. The technology should be validated for varying water matrices to comprehensively assess the cost and environmental impacts associated with these technologies from energy consumption, kinetics, and life cycle perspectives. Further evolution of the enhanced photodissociation of PFAS technology may combine photo- and electrochemical systems for in-situ mineralization techniques. Moreover, the intermediate and free radical nature generated from the photodissociation methods still needs to be fully understood.. Investigation of reaction intermediates for fate, transport, ecotoxicity, and health risk should be conducted. This review is intended to provide researchers in this field a better understanding of recent advances and stimulate further discussions on the photodegradation of perfluorinated compounds.

## Figures and Tables

**Table 1:** Summary of different non-catalytic photodegradative approaches for perfluorinated compounds (PFCs) mineralization.

Technology type	PFAS Type and Concentration (unit varies)	Advantages	Photodegradation Performance and Conditions	Application Scale	Ref.

Photo-oxidation (254 nm UV, 200 W xenon-mercury lamp)	PFOA (C_0_= 1.35 mM)	PFOA directly excited by UV light	89.5% at pH=3.7 72 h of UV irradiation	Bench	[52]
Photo-oxidation (185 nm VUV, 15 W xenon-mercury lamp)	PFOA (C_0_= 25 ppm)	PFOA directly excited by VUV light	61.7% at pH=3.7 2 h of irradiation	Bench	[54]
Photo-oxidation (254 nm UV, 32 W low-pressure mercury lamp)	PFOS (C_0_= 40 μM) NaOH (C= 90 μM)	In alkaline 2-propanol solution, PFOS showed higher degradation under UV light irradiation	76% within 1 day and 92% within 10 days (In alkaline 2-propanol). 8% within 1 day and 68% within 10 days (In water)	Bench	[55]
Photo-oxidation (254 nm UV, 32 W low-pressure mercury lamp; 185 nm VUV) with synthetic and fused silica glass lamp sleeves	PFOA (C_0_= 2.42 μM)	PFOA was more easily decomposed by 185 nm VUV than by UV (254 nm). PFOA degradation and defluorination rate with synthetic fused silica glass sleeve were up to 71-folds larger compared to fused silica glass sleeve.	87.3% within 3 h of 185 nm VUV using synthetic fused silica glass lamp sleeve (single-layered). 30.8% within 3 h of 185 nm VUV using fused silica lamp sleeve (double-layered). pH=3	Bench	[62]
Photo-reduction (254 nm UV, 500 W medium-pressure mercury lamp)	PFOS (C_0_= 37.2 μM) Phosphate buffer solution (PBS, 6.0 mM)	Boiling accelerated PFOS photodegradation compared to non-boiling control.	80–98%; 4 h Temperature= 78–100 °C pH=7	Bench	[57]
Photo-reduction (254 nm UV, 500 W medium-pressure mercury lamp)	PFOS (C_0_= 37.2 μM) Phosphate buffer solution (PBS, 6.0 mM)	Hydrated electrons act as reductive species for PFOS photodegradation under UV irradiation	99.8%; 4 h Temperature = 100 °C pH=11.8	Bench	[58]

**Table 2:** Photochemical mediators for the dissociation of PFCs under UV light irradiation.

Type of Technology/composite	Scale	Mineralization protocol	Justification of mineralization treatment	Type of PFCs, concentration, and conditions	Mineralization	Ref.

Persulfate ion (${\text{S}}_{2}{\text{O}}_{8}^{2-}$) Persulfate ion (${\text{S}}_{2}{\text{O}}_{8}^{2-}$)	Bench	Photo-chemical oxidation (254 nm UV, 200 W lamp)	${\text{S}}_{2}{\text{O}}_{8}^{2-}$ produced highly oxidative $\text{SO}4^{•-}$, which decomposed PFOA	PFOA (C_0_=1.35 mM) ${\text{S}}_{2}{\text{O}}_{8}^{2-}$ (50.0 mM in water) pH=2–3, 4.0 h	100%	[81]
Persulfate ion (${\text{S}}_{2}{\text{O}}_{8}^{2-}$) Persulfate ion (${\text{S}}_{2}{\text{O}}_{8}^{2-}$)	Bench	Photo-chemical oxidation (185 nm UV, 23 W lamp)	Under 185 nm irradiation, PFOA was dissociated by $\text{SO}4^{•-}$	PFOA (C_0_=60 μM) ${\text{S}}_{2}{\text{O}}_{8}^{2-}$ (1.5 mM in water) pH=3.7, 2.0 h	87.4%	[82]
Persulfate ion (${\text{S}}_{2}{\text{O}}_{8}^{2-}$) Persulfate ion (${\text{S}}_{2}{\text{O}}_{8}^{2-}$)	Bench	Photo-chemical oxidation (185/254 nm UV, 6 W lamp)	Under 185/254 nm UV, PFAS was effectively degraded in water with persulfate assistance	PFBA, PFOA, PFOS (C_0_=10 mg/L) ${\text{S}}_{2}{\text{O}}_{8}^{2-}$ (5 g/L) 4.0 h	PFBA: 57% PFOA: 80% PFOS: 60%	[85]
Persulfate and Iron (II)	Bench	Photo-chemical oxidation (254 nm UV, 9 W lamp)	A combination of Fe^2+^/UV with persulfate ions enhanced the mineralization of PFOA	PFOA (C_0_=20 M) ${\text{S}}_{2}{\text{O}}_{8}^{2-}$(30 mM in water) Fe2+ (1 mM) pH=5.0, 5.0 h	93.9%	[83]
Sulfite	Bench	Photo-reductive UV/VUV (254/185 nm) low-pressure mercury lamp	Addition of sulfite led to greater decomposition of PFOS	PFOS (C_0_=5000 ppb) ${\text{SO}}_{3}^{2-}$ (10 mM) pH=12, 6 h	-	[86]

**Table 3:** Role of TiO_2_ based nanocomposites as photocatalysts for PFCs mineralization.

Nano-photocatalyst	Synthesis method	Technology type	Treatment concentration (unit varies)	Advantages	Performance (degradation/defluorination) and Conditions	Ref.

Titanate nanotubes	Titanate nanotubes (TNTs) were synthesized by a microwave hydrothermal method	Photodegradation (254 nm UV, 400 W lamp)	PFOA (C_0_=50 mg/L) TNTs (C=0.5–0.25 g/L)	TNTs served as adsorbents to adsorb PFOA and form TNT-PFOA complexes	Defluorination 85% (pH=4), 68% (pH=7), 55% (pH=10), 24 h	[96]
TiO_2_ slurry	Commercial nano-sized TiO_2_ received from Evonik	Photodegradation via Photo-redox and β-scission (315–400 nm, 500 W Fe-halogenide lamp	PFOA (C_0_=4.0 mM) TiO2 (C=0.66 g/L)	TiO_2_ slurry acted as a photocatalyst	30%, 6 h	[97]
TiO2	P25 (TiO_2_) was obtained from Degussa	Photoreduction (254 nm UV, 23 W low-pressure mercury lamp)	PFOA (C_0_=24 μM) P25 (0.5 g/L) Oxalic acid (3 mM)	Oxalic acid acted as a hole-scavenger which significantly accelerated PFOA decomposition under nitrogen atmosphere and UV irradiation	86.7%, 3 h, N_2_ atmosphere, pH=2.47	[98]
MIP-Ag/TiO_2_ NTs	Anodization followed by Impregnation method	Photocatalytic oxidation (254 nm UV irradiation)	PFOA (C_0_= 50 ppm)	The high performance of MIP-Ag/TiO_2_ NTs was attributed to electron traps of Ag nanoparticles, and the footprint cavities created by MIP	91%, 8 h	[99]
Iron and niobium co-doped titanium dioxide (Fe:Nb-TiO_2_)	Temperature-controlled sol-gel method	Photocatalytic oxidation (254 nm UV, 150 W)	PFOA (C_0_=0.1Mm) Fe:Nb-TiO_2_ (C=0.5gL^−1^)	Fe lowered the band gap energy, and Nb worked as an electron trap and prevented the recombination of electron–hole pairs	14.8%, 180 min, pH=4.3	[100]
Transition-metal modified titanium dioxide (Cu-TiO_2_)	Photo-deposition method	Photocatalytic oxidative degradation (254 nm UV irradiation)	PFOA (C_0_=120.8 μM) Cu-TiO_2_ (C=0.5 g/L)	Copper reduced the band gap energy of TiO_2_ and improved the electron trapping	91%, 12 h, pH=5	[105]
Noble metallic nanoparticle modified TiO_2_ (M-TiO_2_, M= Ag, Pt, Pd)	Chemical reduction method	Photocatalytic oxidation (365 nm UV, 125 W)	PFOA (C_0_=144.9 μM) catalyst (C=0.5 g/L)	Ag, Pd, Pt metallic nanoparticles captured electrons more efficiently	57.7–100%, 7 h, pH=3	[101]
Pb-modified titanium dioxide (Pb-TiO2)	Chemical reduction method	Photocatalytic oxidation (254 nm UV, 400 W)	PFOA (C_0_=120.8 μM) catalyst (C=0.5 g/L)	Photocatalysts yields traps to capture photo-induced electrons or holes leading to better photocatalytic efficiencies then pure TiO2	99.9%, 12 h, pH=5	[102]
TiO_2_-MWCNT composite	Sol-gel method	Photocatalytic oxidation (365 nm UV, 300 W)	PFOA (C_0_=72.5 μM) catalyst (C=1.6 g/L)	Adsorption and electron transport capacity of MWCNT enhanced the photocatalytic activity	94% pH=5	[103]

**Table 4:** Role of alternative nano-empowered technologies in photocatalysis for PFCs mineralization.

Nano-photocatalyst	Synthesis method	Technology type	Treatment concentration (unit varies)	Advantages	Performance (degradation/defluorination)	Ref.

In2O3	Commercially available In_2_O_3_	Photooxidative degradation (254 nm UV, 23 W lamp)	PFOA (C_0_=100 μM) Catalyst (C=1.6 g/L)	The terminal carboxylate (-COO^−^) group of PFOA coordinates with In_2_O_3_ in surface bidentate or bridging configuration.	83.1%, 4 h	[108]
In_2_O_3_ nanoporous nanosphere (In_2_O_3_ NPNs)	Solvothermal method followed by calcination	Photooxidative degradation (254 nm UV, W lamp)	PFOA (C_0_=72.5 μM) Catalyst (C=0.50 g/L)	Nanoporous structure and large surface area enhanced the photocatalytic activity of In_2_O_3_	71%, 3 h, pH=3.9	[110]
Nanostructured In_2_O_3_	Solvothermal process followed by calcination	Photooxidative degradation (254 nm UV irradiation, 15 W lamp)	PFOA (C_0_=72.5 μM) Catalyst (C=0.50 g/L)	Nano-structured In_2_O_3_ enhanced the electron transfer between catalyst and PFOA	100%, 0.5–2 h pH=3.9	[111]
In_2_O_3_-graphene composites	sonication-followed by calcination	Photooxidative degradation (254 nm UV irradiation, 15 W lamp)	PFOA (C_0_=72.5 μM) Catalyst (C=0.50 g/L)	Absorbance performance of graphene and unprotected surface of In_2_O_3_ impacted PFOA dissociation	87%, 3 h	[112]
CeO2-doped indium oxide (xCeO_2_/In_2_O_3_)	Solvothermal method followed by calcination	Photooxidative degradation (254 nm UV)	PFOA (C_0_=100 mg/L)	Effective inhibition of recombination of photo-induced electron-holes caused by the charge transfer between CeO_2_ and In_2_O_3_, enhanced the dissociation of PFOA	*<* 90%,1 h, pH=4.6	[113]
Nanostructured gallium oxide (Ga_2_O_3_)	Hydrothermal method followed by calcination	Photo-reductive degradation (254 nm UV, 14 W lamp	PFOA (C_0_=500 μg/L) Catalyst (C=0.50 g/L)	Surfacearea ofthe Nanostructured β-Ga_2_O_3_ with a needle-like structure provides more adsorption and reaction sites	Approx. 100% at pH=4.8, *<* 1-hour reaction	[114]
SiC/graphene	Ultrasonication followed by Hydrothermal method	Photo-reductive degradation (254 nm UV)	PFOA (C_0_=0.12 mmol/L) Catalyst (C=0.50 g/L)	Reactive Si-H bonds can be generated on SiC/graphene under UV light excitation, substituting for silylium (R3Si+ ) to activate C− F bonds. The decomposition of PFOA was attributed to the hydrodefluorination process via the Si− H/C− F redistribution	40.5–58.5%, 8 h, pH=7	[115]
Graphene quantum dots attached to SiC nanoparticles (SiC/GQDs)	Oxygen-driven unzipping of graphene under ultra-high frequency ultrasonication, and then attached to the SiC nanoparticles by the hydrothermal method	Photocatalytic degradation (254 nm UV)	PFOS (C_0_=0.019 mM)	GQDs designed the heterojunction with SiC and were the electron donor; providing robust reductive potential to mineralize PFOS	88.5%,20 h, pH=7	[116]
Platinum modified indium oxide nanorods (Pt/IONRs)	Solvothermal method followed by photo-deposition method	Photooxidative degradation (254 nm UV, 15 W lamp)	PFOA (C_0_=200 mg/L) Catalyst (C=0.50 g/L)	Rod like-structure of IONRs and Pt-doping increased the UV light harvesting; leading to photogenerated holes and ${\text{O}}_{2}{•}^{-}$ which played an important role in PFOA degradation	98–5%, l h pH=1.8–9.27	[117]
